# Antiproliferative Role of Natural and Semi-Synthetic Tocopherols on Colorectal Cancer Cells Overexpressing the Estrogen Receptor β

**DOI:** 10.3390/ijms26052305

**Published:** 2025-03-05

**Authors:** Irene Falsetti, Gaia Palmini, Roberto Zonefrati, Kristian Vasa, Simone Donati, Cinzia Aurilia, Allegra Baroncelli, Caterina Viglianisi, Francesco Ranaldi, Teresa Iantomasi, Piero Procacci, Stefano Menichetti, Maria Luisa Brandi

**Affiliations:** 1 Department of Experimental and Clinical Biomedical Sciences “Mario Serio”, University of Florence, Viale Pieraccini 6, 50139 Firenze, Italy; irene.falsetti@unifi.it (I.F.); simone.donati@unifi.it (S.D.); cinzia.aurilia@unifi.it (C.A.); francesco.ranaldi@unifi.it (F.R.); 2 Italian Foundation for Research on Bone Disease (F.I.R.M.O.), Via San Gallo 123, 50129 Firenze, Italy; gaia@fondazionefirmo.com (G.P.); marialuisa@marialuisabrandi.it (M.L.B.); 3 Department of Chemistry “Ugo Schiff”, University of Florence, Via Della Lastruccia, 3–13, 50019 Sesto Fiorentino, Italy; kristian.vasa@unifi.it (K.V.); allegra.baroncelli@edu.unifi.it (A.B.); caterina.viglianisi@unifi.it (C.V.); piero.procacci@unifi.it (P.P.); stefano.menichetti@unifi.it (S.M.)

**Keywords:** estrogen receptor β, colorectal cancer, δ-tocopherol, δ-tocopherol sulfide/disulfide, cell proliferation, reactive oxygen species

## Abstract

Estrogen receptor β (ERβ) is the most highly expressed subtype in the colon epithelium and mediates the protective effect of estrogen against the development of colon cancer. Indeed, the expression of this receptor is inversely related to colorectal cancer progression. Structurally estrogen-like compounds, including vitamin E components, affect cell growth by binding to ERs. In the present study, cell proliferation was measured by cell counting in a Bürker hemocytometer, and ERβ expression was measured by Real-Time qPCR and immunoenzymatic methods. The results obtained show that natural δ-tocopherol (**δ-Toc**) and two of its semi-synthetic derivatives, bis-δ-tocopheryl sulfide **(δ-Toc)_2_S** and bis-δ-tocopheryl disulfide **(δ-Toc)_2_S_2_**, play an antiproliferative role and upregulate ERβ expression, similar to 17-β-estradiol (17β-E2), in human colon adenocarcinoma HCT8 cells engineered to overexpress ERβ protein (HCT8-β8). These events are not present in HCT8-pSV2neo and in HCT8-β8 pretreated with ICI 182,780, suggesting that they are mediated by the binding of compounds to ERβ, as also boosted by an in silico assay. The antiproliferative effect is independent of the intracellular redox state and **(δ-Toc)_2_S** and **(δ-Toc)_2_S_2_** reduce cell proliferation at concentrations lower than that of **δ-Toc** and all tested compounds are also able to upregulate ERβ expression. Taken together, the data indicate that, through the involvement of ERβ activity and expression, **δ-Toc**, **(δ-Toc)_2_S**, and **(δ-Toc)_2_S_2_** may provide potential therapeutic support against colorectal cancer.

## 1. Introduction

Estrogen receptors (ERs), including ERα and ERβ, belong to the nuclear receptor family and have a similar structure; both receptor subtypes, after binding to estrogen, induce the transcription of estrogen-responsive genes [1]. However, the biological functions of ERα and ERβ are different as well as their tissue targets [2]. Generally, ERα shows proliferative and anti-apoptotic effects, whereas ERβ exerts antiproliferative and pro-apoptotic roles [3,4,5,6]. ERα is very poorly expressed in the colon, where ERβ is highly expressed in both normal and malignant epithelium, although ERβ expression is inversely associated with colorectal cancer (CRC) progression [7,8]. The advanced stage and grade of colon cancer is associated with a decreased ERβ expression, which is responsible for the increased proliferation and decreased differentiation and apoptosis in colon cancer cells [9,10]. Given that CRC affects more men than women and the incidence is reduced by hormone replacement therapy in ERβ-positive postmenopausal women [11], a protective role in the prevention and/or progression of CRC has been attributed to estrogen and ERβ [10,12,13]. Estrogen exerts its protective role in CRC through the involvement of ERβ [14,15,16,17], and the activation and/or increased expression of ERβ reduce intestinal tumorigenesis in animal models [18,19,20]. Reduced cell growth occurs in ERβ-transfected colon cancer cells and their stimulation with phenolic compounds; having a structure similar to that of 17-β-estradiol (17β-E2) induces an upregulation of ERβ expression and downregulation of cell proliferation and viability [21,22,23,24].

Vitamin E is the fat-soluble, most antioxidant compound found in humans. It consists of eight different molecules, specifically, α-, β-, γ-, and δ-tocopherol (α-, β-, γ-, and **δ-Toc**) and α-, β-, γ-, and δ-tocotrienol [25]. Due to its antioxidant capacity, vitamin E may have a beneficial effect on diseases associated with oxidative stress, such as inflammatory, neurodegenerative, and cardiovascular diseases [26,27,28]. However, in addition to its antioxidant activity, vitamin E is also able to regulate the cell cycle, apoptosis, and cell proliferation by influencing the activity of a number of enzymes, the activation of transcription factors, and signal transduction pathways [25,29]. It has been shown that natural forms and synthetic derivatives of vitamin E have an antiproliferative effect in cancer cells but not in normal cells [30,31,32,33,34].

Due to the presence of a phenolic group in the structure, vitamin E compounds can influence cell proliferation by binding to ERs. In fact, both tocopherols and tocotrienols interact with ERs, even if δ isoforms appear to have a higher affinity than α and γ. Then, they can modulate the ER-dependent gene expression [35,36]. Tocotrienols have been shown to have a high affinity for ERβ, but not for ERα, and to exert antiproliferative and pro-apoptotic effects through the nuclear translocation of ERβ and the expression of pro-apoptotic estrogen-responsive genes in ERβ-expressing breast cancer MDA-MB-231 cells and in human breast cancer MCF-7 cells expressing both ERα and ERβ [37,38]. Tocopherols inhibit cell invasion in ER-positive breast cancer, while a derivative of **α-Toc**, unlike **α-Toc**, can reduce MCF-7 cell proliferation [39,40]. Although it has been shown that **γ**- and **δ-Toc**, but not **α-Toc**, inhibit colon carcinogenesis [41,42], to our knowledge, no data have been reported in the literature on the role of vitamin E components on ER-mediated cell proliferation in colon cancer. Therefore, the aim of this study was to evaluate the role of natural **δ-Toc** and two of its semi-synthetic derivatives, bis-δ-tocopheryl sulfide **(δ-Toc)_2_S** and bis-δ-tocopheryl disulfide **(δ-Toc)_2_S_2_**, on ERβ-mediated cell proliferation and the regulation of ERβ expression in human colon adenocarcinoma HCT8 cells transfected with the plasmid vector pCXN2-hERβ for the overexpression of ERβ (HCT8-β8). Semi-synthetic derivatives of **δ-Toc** containing sulfide and disulfide bonds were tested, as the intrinsic ability of a ligand to activate a receptor to induce the receptor-mediated cellular response is also related to its chemical structure [43]. In addition, they have previously been shown to exhibit differences in chain-breaking antioxidant activity in vitro [44]**,** and may be differently affected by the intracellular redox state and, thus, differentially influence potential redox-mediated biological processes. The use of HCT8-β8 allowed the in vitro evaluation of the role of ERβ in colon tumorigenesis and how this can be regulated by molecules, structural analogues of 17β-E2, through their binding to the estrogen receptor. To clarify the actual involvement of ERβ in the role of the tested compounds, experiments were also performed in the presence of 17β-E2 and in HCT8 cells transfected with the control pSV2neo vector (HCT8-pSV2neo).

## 2. Results

**Figure 1:**
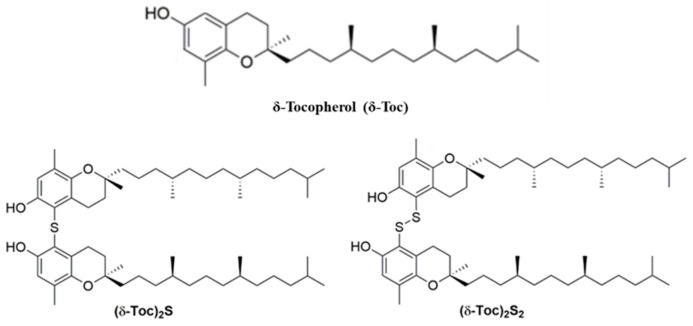
Structure of δ-tocopherol, **δ-Toc**, bis-δ-tocopheryl sulfide, **(δ-Toc)_2_S**, and bis- δ-tocopheryl disulfide, **(δ-Toc)_2_S_2_**.

The experiments were performed in medium containing 0.5% charcoal-stripped fetal bovine serum (FBS) (starved medium) to deplete steroids, and the range of concentrations used for **δ-Toc**, **(δ-Toc)_2_S,** and **(δ-Toc)_2_S_2_** also includes those that have been reported in the literature for vitamin E compounds [36,39]. However, in our experiments, the normal concentration (μN) was used for the tocopherol-containing compounds because **(δ-Toc)_2_S** and **(δ-Toc)_2_S_2_** have two δ-tocopherol units in their structures compared to **δ-Toc** (Figure 1).

In this way, we can compare the effects of the natural **δ-Toc** and the semi-synthetic derivatives, considering the same concentration of tocopherol units. Consequently, the molarity of the **(δ-Toc)_2_S** and **(δ-Toc)_2_S_2_** is half that of the **δ-Toc**. Then, for **δ-Toc**, the concentrations 12.5, 25, 50, 100, and 250 μN are equivalent to the same concentrations expressed in μM. On the contrary, for semi-synthetic **(δ-Toc)_2_S** and **(δ-Toc)_2_S_2_**, the concentrations 12.5, 25, 50, 100, and 250 μN correspond to 6.25, 12.5, 25, 50, and 125 μM. In order to have uniformity in the concentrations, we preferred to use the normal concentration.

### 2.1. Effect of 17β-E2, Natural δ-Toc, and Semi-Synthetic (δ-Toc)_2_S and (δ-Toc)_2_S_2_ on Proliferation of HCT8-β8 Cells, Treated or Not with ICI 182,780, and in HCT8-pSV2neo Cells

**Figure 2:**
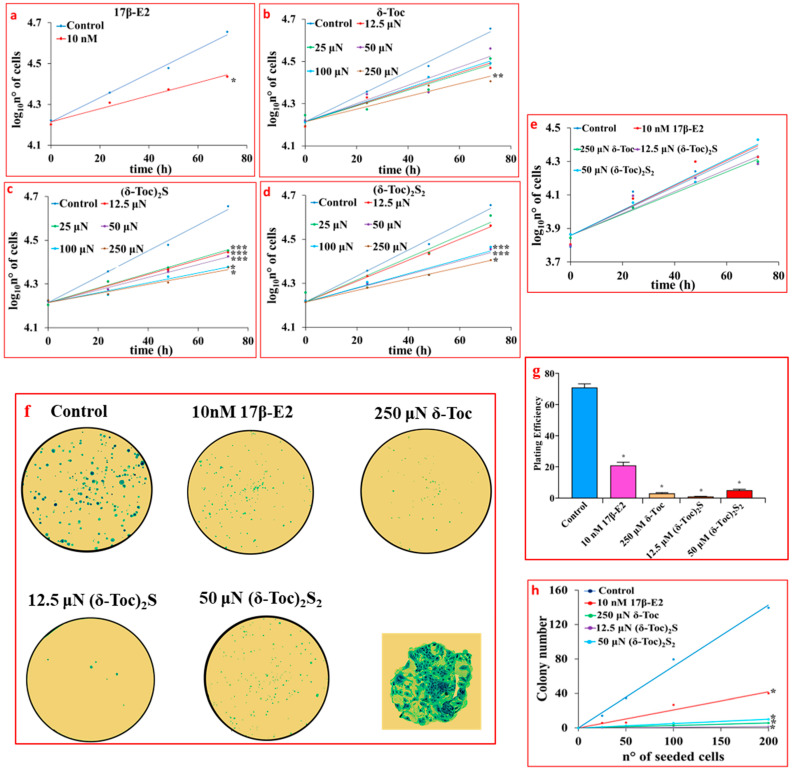
Proliferation (**a**–**e**) and colony formation (**f**–**h**) assays in cells treated with 17β-E2, natural **δ-Toc**, or semi-synthetic **(δ-Toc)_2_S** and **(δ-Toc)_2_S_2_**. HCT8-β8 and HCT8-pSV2neo cells were cultured in charcoal-stripped 0.5% FBS and untreated (control) or treated with 10 nM 17β-E2 or concentrations in the range (12.5–250 μN) of **δ-Toc**, **(δ-Toc)_2_S**, or **(δ-Toc)_2_S_2_** for 0, 24, 48, and 72 h to detect proliferation, as described in Section 4. The same treatments were performed for 10 days to detect colony formation in HCT8-β8. (**a**–**e**) Proliferation values are the ean ± SE of three experiments repeated in tetraplicate and are expressed as the log_10_ of cell number. (**f**) Representative colony formation images in 200 cells/well; amplification of a representative colony is shown in the square. (**g**) Plating efficiency values in 200 cell/well are the ratio between number of colonies formed and number of cells seeded × 100, (**h**) Colony number relative to number of cells seeded (25–200 cells). Plating efficiency and colony number values are the mean ± SE of three independent experiments. * *p* ≤ 0.001; ** *p* ≤ 0.01; *** *p* ≤ 0.05 compared to control.

Figure 2a shows that 17β-E2, used as a positive control for an antiproliferative effect, significantly reduced the proliferation of HCT8-β8 (94 ± 20 h for the population-doubling time) compared to untreated cells (51 ± 10 h for the population-doubling time) with an inhibitory effect of 76%. **δ-Toc, (δ-Toc)_2_S**, and **(δ-Toc)_2_S_2_** were shown to have an antiproliferative effect similar to that of estrogen. In particular, the antiproliferative effect of sulfur derivatives appeared to be superior to that of **δ-Toc**, as only the highest concentration (250 μN) of **δ-Toc** was able to significantly reduce HCT8-β8 proliferation (100 ± 23 h for the population-doubling time with a 96% inhibition) (Figure 2b). All concentrations of **(δ-Toc)_2_S** used significantly inhibited proliferation with 12.5 μN as the minimum responsive concentration (93 ± 16 h for the population-doubling time with an inhibition of 82%) (Figure 2c), while the antiproliferative effect of **(δ-Toc)_2_S_2_** was evident from the concentration of 50 µN with a population-doubling time of 96 ± 21 h with an inhibition of 88% (Figure 2d). However, it should be noted that the sulfur derivatives did not show a concentration-dependent effect in the responsive concentration range. In fact, the antiproliferative effect was not significantly different among the different effective concentrations (Figure 2c,d).

Taking all this into account, in the following experiments, **δ-Toc** was used at 250 μN, which is the only effective concentration, while the semi-synthetic compounds, **(δ-Toc)_2_S** and **(δ-Toc)_2_S_2_**, were tested at the lowest concentrations at which they were effective (12.5 and 50 µN, respectively).

Figure 2e shows that the proliferation of HCT8-pSV2neo cells was not affected by 10 nM 17β-E2, 250 μN **δ-Toc**, 12.5 μN **(δ-Toc)_2_S**, and 50 μN **(δ-Toc)_2_S_2_**. These treatments were carried out in HCT8-β8 in order to confirm the antiproliferative role of the tocopherol-containing compounds by means of the colony formation assay. The results obtained show that, when 200 cells were seeded in 6-well plates and treated with 17β-E2, **δ-Toc**, **(δ-Toc)_2_S**, and **(δ-Toc)_2_S_2_** for 10 days, the number of colonies was very, very low compared to the control (Figure 2f,g). In addition, the number of HCT8-β8 colonies was dependent on the different number of cells seeded in the range of 25–200 cells/well and was reduced by treatments compared to control (Figure 2h). In the presence of treatments, the number of colonies in HCT8-pSV2neo was not different from that in controls. 

**Figure 3:**
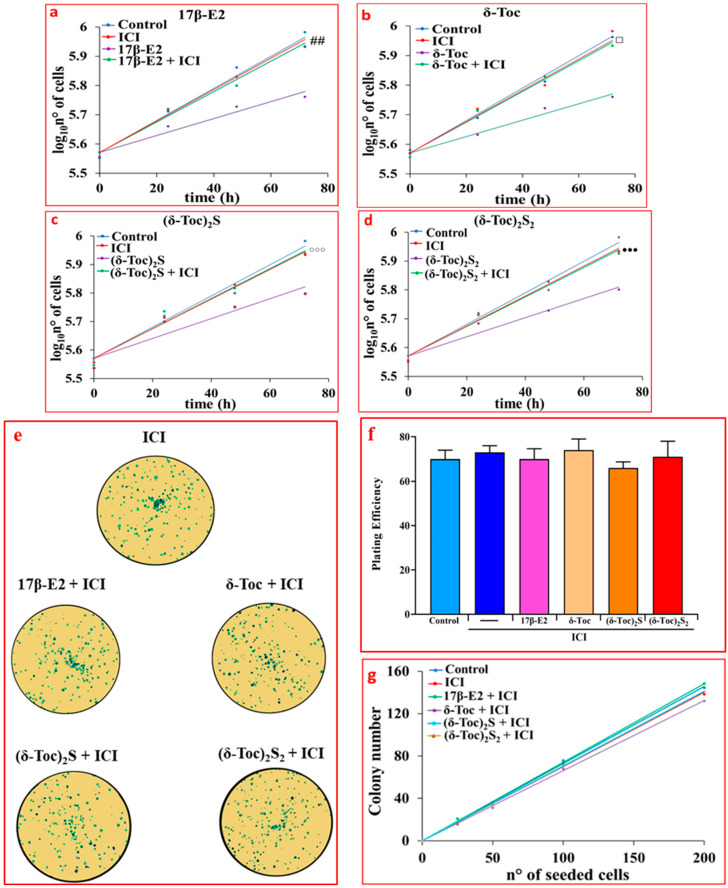
Proliferation (**a**–**d**) and colony formation assay (**e**–**g**) of HCT8-β8 treated with 17β-E2, natural **δ-Toc,** or semi-synthetic **(δ-Toc)_2_S** and **(δ-Toc)_2_S_2_**, in the presence of ICI 182,780. HCT8-β8 cells, cultured in 0.5% charcoal-stripped FBS were pretreated for 1 h with 10 μM ICI 182,780 (ICI) and untreated (control) or treated with 10 nM 17β-E2, 250 μN **δ-Toc**, 12.5 μN **(δ-Toc)_2_S**, or 50 μN (**δ-Toc)_2_S_2_** for 0, 24, 48, and 72 h to detect proliferation or for 10 days to detect colony formation, as described in Section 4. (**a**–**d**) Proliferation values are the mean ± SE of three experiments repeated in tetraplicate and are expressed as the log_10_ of cell number; (**e**) Representative colony formation images in 200 cells/well. (**f**) Plating efficiency values in 200 cell/well are the ratio between number of colonies formed and number of cells seeded × 100, (**g**) Colony number relative to number of cells seeded (25–200 cells). Plating efficiency and colony number values are the mean ± SE of three independent experiments. ## *p* ≤ 0.01 compared to 17β-E2; ⸋ *p* ≤ 0.001 compared to **δ-Toc**; ⸰⸰⸰ *p* ≤ 0.05 compared to **(δ-Toc)_2_S**; ^●●●^ *p* ≤ 0.05 compared to **(δ-Toc)_2_S_2_**.

To assess the involvement of ERβ in the antiproliferative role of tocopherol-containing compounds, the proliferation and colony formation were investigated in HCT8-β8 pretreated for 1 h with 10 µM ICI 182,780 (fulvestrant) to which were added 10 nM 17β-E2, 250 μN **δ-Toc**, 12.5 μN **(δ-Toc)_2_S,** or 50 μN **(δ-Toc)_2_S_2_**. ICI 182,780, an estrogen-receptor-signaling antagonist capable of binding to ERs, causes the alteration of receptor dimerization and increases receptor degradation [45,46]. The results show that ICI 182,780 prevented the antiproliferative effect of 17β-E2 (Figure 3a) and tocopherol-containing compounds (Figure 3b–d), suggesting the involvement of ERβ in their effect on proliferation and a possible binding of these compounds to the receptor. In the presence of ICI 182,780, the number of colonies was similar in all conditions (Figure 3e–g).

### 2.2. Effect of 17β-E2, Natural δ-Toc, and Semi-Synthetic (δ-Toc)_2_S and (δ-Toc)_2_S_2_ on Viability of HCT8-β8 and HCT8-pSV2neo Cells

**Figure 4:**
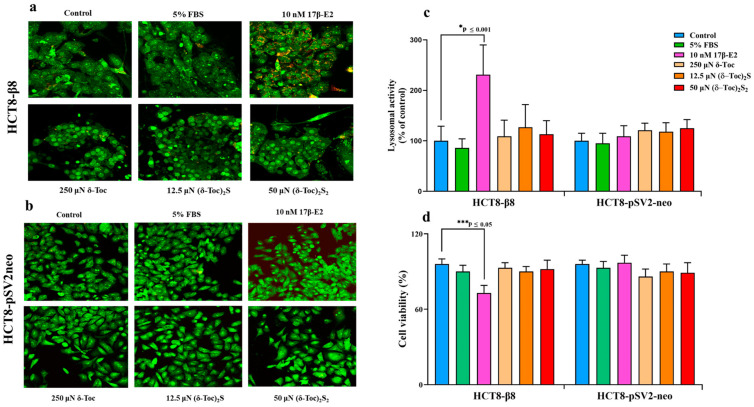
Microscopy observation (**a**,**b**), quantitative evaluation of lysosomal activity (**c**), and trypan blue dye exclusion test (**d**) in HCT8-β8 and HCT8-pSV2neo cells treated with 17β-E2, natural **δ-Toc**, or semi-synthetic **(δ-Toc)_2_S** and **(δ-Toc)_2_S_2_**. HCT8-β8 and HCT8-pSV2neo cells were cultured in 5% FBS; or in 0.5% charcoal-stripped FBS and untreated (control) or treated with 17β-E2, **δ-Toc**, **(δ-Toc)_2_S,** or **(δ-Toc)_2_S_2_** for 72 h and stained with acridine orange (AO) or trypan blue dye as described in Section 4. (**a**,**b**) Representative image from three independent experiments. Fluorescence measured with LSM 900 confocal microscopy shows lysosomes in red-orange (λ_ex_ 405 nm/λ_em_ 575–700 nm), and nuclei and mitochondria in green (λ_ex_ 488 nm/λ_em_ 495–560 nm); original magnification: 40×. (**c**) Quantitative values of lysosomal activity, expressed as red/green fluorescence ratio and reported as percentage of control, are the means ± SD of three experiments. (**d**) The percentage of viable cells was calculated as the ratio of unstained cells to total cells × 100. Values are the means ± SD of three experiments repeated in triplicate.

Cell viability was determined using acridine orange (AO) staining to detect the lysosome formation, an indicator of cell death, and trypan blue dye which is only taken up by dead cells. In fact, an increased lysosome formation and activation is an early sign of cell distress leading to subsequent apoptosis and cell death [47], whereas the trypan blue dye exclusion test is based on the staining of dead or damaged cells with compromised cell membranes [48]. The lysosomal fluorescence staining at 72 h (Figure 4a,b) and the relative lysosomal activity (Figure 4c) detected in HCT8-β8 and HCT8-pSV2neo are shown. In particular, HCT8-β8 cells treated with 10 nM 17β-E2 showed a significant increase in lysosomal activity, resulting in a decrease in viability at all times tested, with no differences between the different times; on the contrary, **δ-Toc**, **(δ-Toc)_2_S,** and **(δ-Toc)_2_S_2_**, tested at the lowest responsive concentrations for proliferative activity (250, 12.5, and 50 μN for **δ-Toc**, **(δ-Toc)_2_S,** and **(δ-Toc)_2_S_2_**, respectively), did not cause a significant change in viability compared to the control (Figure 4a–c). Results at 24 h and 48 h were similar to those at 72 h. The trypan blue dye exclusion assay performed at 72 h confirmed these results and showed that only 17β-E2 significantly reduced HCT8-β8 viability by approximately 25% compared to control cells (Figure 4d). The lysosomal activity and trypan blue assay showed that the viability of HCT8-pSV2neo cells was not affected by either 17β-E2 or tocopherol-containing compounds (Figure 4b–d).

### 2.3. Effect of 17β-E2, Natural δ-Toc, and Semi-Synthetic (δ-Toc)_2_S and (δ-Toc)_2_S_2_ on Apoptosis and Expression of CCND1 and PLK1 Genes in HCT8-β8 Cells

**Figure 5:**
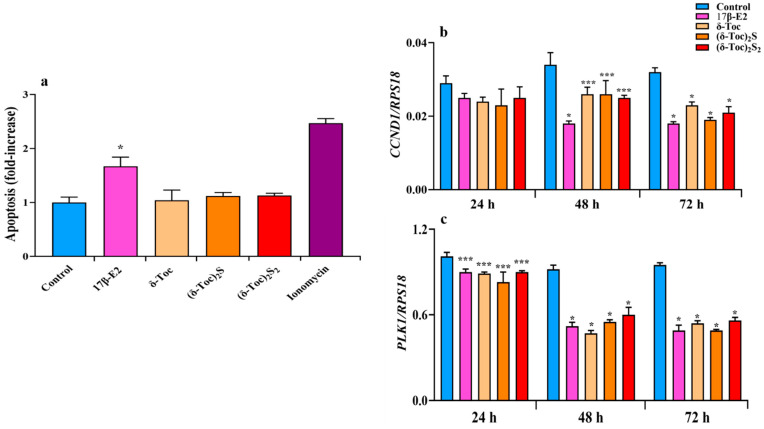
Apoptosis (**a**) and expression of *CCND1* (**b**) and *PLK1* (**c**) genes in HCT8-β8 cells treated with 17β-E2, natural **δ-Toc,** or semi-synthetic **(δ-Toc)_2_S**, **(δ-Toc)_2_S_2_** and ionomycin. HCT8-β8 cells were cultured in 0.5% charcoal-stripped FBS and untreated (control) or treated with 10 nM 17β-E2, 250 μN **δ-Toc**, 12.5 μN **(δ-Toc)_2_S**, 50 μN **(δ-Toc)_2_S_2_**, or 1 μM ionomycin for 72 h to detect apoptosis or for 24, 48, and 72 h to detect *CCND1* and *PLK1* genes’ expression, as described in Section 4. (**a**) Apoptosis data, relative to mono- and oligonucleosomes released into the cytoplasmic fraction from 10^4^ cells, are expressed as fold increase over the control value, using the absorbance of sample/absorbance of control ratio, and are the mean ± SD of three experiments repeated in triplicate. (**b**,**c**) Quantitative real-time qPCR analysis of *CCND1* and *PLK1* was performed and the values, expressed as the number of mRNA molecules of the gene and normalized to ribosomal protein S1 (RPS18) housekeeping gene (*RPS18*) mRNA, are the mean ± SD of three experiments repeated in triplicate. * *p* ≤ 0.001; *** *p* ≤ 0.05 compared to respective controls.

In HCT8-β8 cells after 72 h of treatment, an apoptosis analysis shows that **δ-Toc** and its sulfur derivatives at the concentrations tested did not induce apoptotic death unlike 17β-E2, which caused a significant apoptotic effect (Figure 5a). These results were in line with the viability results; 1 μM ionomycin, a calcium ionophore that increases intracellular calcium levels and promotes apoptosis [49], was used as a positive control for apoptosis.

To better understand the antiproliferative role of the compounds tested, we also analyzed the regulation of the cell cycle by evaluating the expression of genes such as cyclin D (CCDN1), which encodes the cyclin D1 involved in the transition from G1 to S phase, and polo-like kinase 1 (PLK1), which encodes the PLK1 critical for the G2/M transition and mitosis [50,51]. After the treatment of HCT8-β8 with estrogen- and tocopherol-containing compounds for 48 and 72 h, both CCDN1 and PLK1 expression were significantly decreased compared to control (Figure 5b,c). After 24 h, all treatments reduced CCND1 and PLK1 expression, but only the PLK1 reduction was significant (Figure 5b,c).

### 2.4. Effect of 17β-E2, Natural δ-Toc, and Semi-Synthetic (δ-Toc)_2_S and (δ-Toc)_2_S_2_ on ERβ and ERα Expression in HCT8-β8 and HCT8-pSV2neo Cells

**Figure 6:**
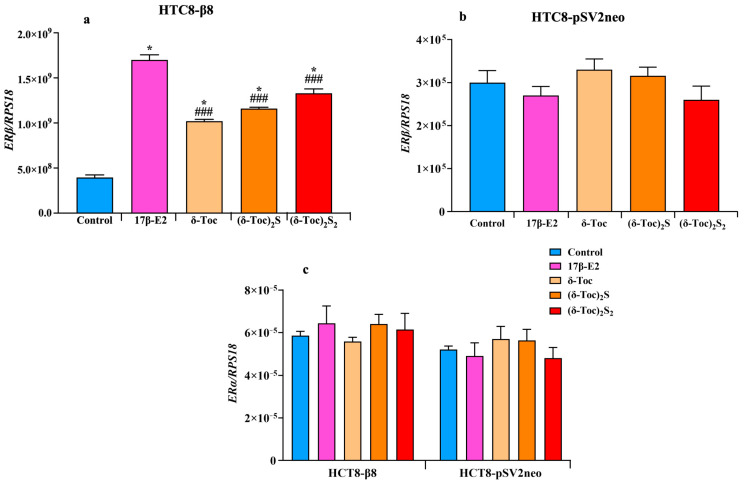
Real-time qPCR analysis of *ERβ* (**a**,**b**) or *ERα* (**c**) genes’ expression in HCT8-β8 and HCT8-pSV2neo cells treated with 17β-E2, natural **δ-Toc,** or semi-synthetic **(δ-Toc)_2_S** and **(δ-Toc)_2_S_2_**. HCT8-β8 and HCT8-pSV2neo cells were cultured in 0.5% charcoal-stripped FBS and untreated (control) or treated with 10 nM 17β-E2, 250 µN **δ-Toc**, 12.5 µN **(δ-Toc)_2_S,** or 50 µN **(δ-Toc)_2_S_2_** for 24 h as described in Section 4. Quantitative real-time qPCR analysis of the *ERβ* (**a**,**b**) and *ERα* (**c**) expression was performed and the values, expressed as the number of mRNA molecules of the gene and normalized to ribosomal protein S1 (RPS18) housekeeping gene (RPS18) mRNA, are the mean ± SD of three experiments repeated in tetraplicate. * *p* ≤ 0.001 compared to control; ^###^ *p* ≤ 0.05 compared to 17β-E2.

Figure 6a shows that the expression of ERβ in HCT8-β8, treated or not with 10 nM 17β-E2, 250 μN **δ-Toc,** 12.5 μN **(δ-Toc)S_2_**, or 50 μN **(δ-Toc)_2_S_2_**, was significantly upregulated by both 17β-E2 and all tocopherol-containing compounds as compared to untreated cells. However, with 17β-E2, the levels of ERβ expression were significantly higher than those obtained with the tocopherol-containing compounds, whose ERβ mRNA levels were similar and not significantly different from each other (Figure 6a). On the contrary, no change in ERα expression levels was detected in the presence of the treatments (Figure 6c). In HCT8-pSVneo treated with 17β-E2, **δ-Toc**, **(δ-Toc)S_2_**, or **(δ-Toc)_2_S_2_**, the expression of *ERβ* (Figure 6b) and *ERα* (Figure 6c) did not change compared to control cells.

### 2.5. Effect of 17β-E2, Natural δ-Toc, and Semi-Synthetic (δ-Toc)_2_S and (δ-Toc)_2_S_2_ on Protein ERβ Expression in HCT8-β8 Cells

**Figure 7:**
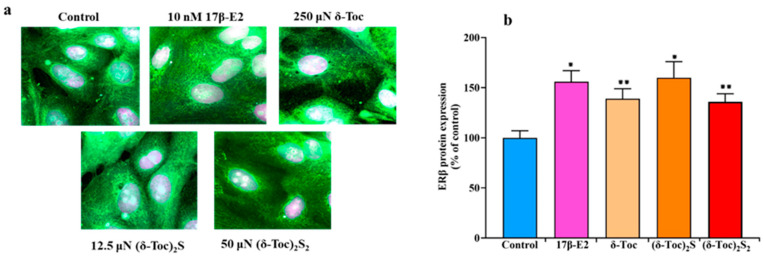
Immunofluorescence staining (**a**) and protein levels (**b**) of ERβ in HCT8-β8 treated with 17β-E2, natural **δ-Toc,** or semi-synthetic **(δ-Toc)_2_S** and **(δ-Toc)_2_S_2_**. HCT8-β8 cells, cultured in 0.5% charcoal-stripped FBS, were untreated (control) or treated with 10 nM 17β-E2, 250 µN **δ-Toc**, 12.5 µN **(δ-Toc)_2_S,** or 50 µN **(δ-Toc)_2_S_2_** for 48 h as described in Section 4. (**a**) Representative image from three independent experiments. Immunofluorescence staining was performed with Alexa Fluor 488 and propidium iodide. Fluorescence was measured by LSM confocal microscopy in conventional colors: green for ERβ (Alexa Fluor 488) and violet (propidium iodide) for nuclei. Original magnification: 63×. (**b**) ERβ protein levels were assayed by ELISA kit and the values, normalized on total protein content and expressed as the percentage of control, are the mean ± SD of three experiments repeated in tetraplicate. * *p* ≤ 0.001 compared to control; ** *p* ≤ 0.01 compared to control.

To demonstrate the presence of ERβ and the validity of our cellular model, ERβ was assessed by immunofluorescence staining in HCT8-β8 treated or not with 10 nM 17β-E2, 250 µN **δ-Toc**, 12.5 µN **(δ-Toc)_2_S,** and 50 µN **(δ-Toc)_2_S_2_** for 48 and 72 h. A microscopic observation at 48 h of HCT8-β8 showed the presence of ERβ for the control and all the treatments (Figure 7a). The results at 72 h were similar. However, because it is difficult to detect possible variations in ERβ levels under different treatments by immunofluorescence assay, the levels of ERβ under the same experimental conditions were detected by an immunoenzymatic method. Figure 7b shows that the levels of ERβ protein expression were similarly increased by 17β-E2 and all tocopherol-containing compounds.

### 2.6. Intracellular Redox State During Proliferation of HCT8-β8 Treated or Not with 17β-E2, Natural δ-Toc, and Semi-Synthetic (δ-Toc)_2_S, (δ-Toc)_2_S_2_

**Figure 8:**
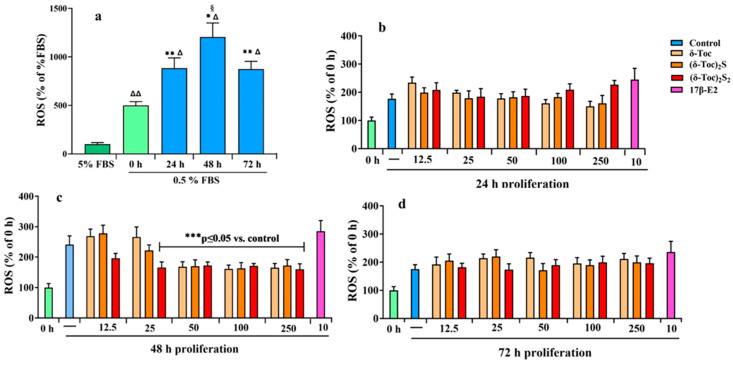
Intracellular ROS production in HCT8-β8 untreated or treated with 17β-E2, natural **δ-Toc**, or semi-synthetic **(δ-Toc)_2_S** and **(δ-Toc)_2_S_2_** during proliferation. ROS levels were detected by measuring the fluorescence intensity of the intracellular-oxidation-sensitive probe H2DCFDA. (**a**) HCT8-β8 cells were cultured in 5% FBS or 0.5% charcoal-stripped FBS (0.5% FBS) for 0 (control), 24, 48, and 72 h as described in Section 4. The values, normalized on total protein content and expressed as percentage of 5% FBS, are the mean ± SD of three experiments repeated in tetraplicate. ^Δ^ *p* ≤ 0.001; ^ΔΔ^ *p* ≤ 0.01 compared to 5% FBS; * *p* ≤ 0.001; ** *p* ≤ 0.01 compared to control (0 h); ^§^ *p* ≤ 0.05 compared to 24 and 72 h. (**b**–**d**) HCT8-β8 cells were cultured in 0.5% charcoal-stripped FBS for 0 h and untreated (—, control) or treated with 10 nM 17β-E2 or various concentrations (12.5–250 μN) of tocopherol-containing compounds for 24, 48, and 72 h as described in Section 4. The values, normalized on total protein content and expressed as percentage of control, are the mean ± SD of three experiments repeated in tetraplicate. *** p ≤ 0.05 compared to control.

Nutrient deprivation, such as serum starvation, promotes alterations of mitochon-drial function in cancer cells, resulting in reduced ATP production and increased reactive oxygen species (ROS) levels [52]. In fact, mitochondria are the main source of ROS and a link has been shown between starvation and mitochondrial ROS modulator 1, which is involved in ROS production [53]. Thus, firstly, the intracellular redox state in HCT8-β8 cells was determined by measuring ROS levels in untreated cells cultured in a complete medium (5% FBS) and in a starved medium during proliferation. Figure 8a shows that starvation induced oxidative stress; indeed, ROS levels progressively increased from time 0 to 48 h of proliferation compared to the levels measured in cells grown with 5% FBS. After 72 h of proliferation, ROS levels remained high but decreased to the 24 h levels.

Subsequently, starved cells were treated for 24, 48, and 72 h with 10 nM 17β-E2 and tocopherol-containing compounds at all concentrations (12.5–250 μN) used to detect the proliferation. Figure 8a–d shows that ROS levels in cells treated with 17β-E2 were similar to those in untreated cells (control) at all times. Even with **δ-Toc**, **(δ-Toc)_2_S,** and **(δ-Toc)_2_S_2_**, ROS levels at all concentrations were similar to their respective controls after 24 and 72 h of proliferation (Figure 8b,d). After 48 h of proliferation, the ROS levels were similar to untreated cells with all tocopherol-containing compounds at 12.5 μN and with 25 μN **δ-Toc** and **(δ-Toc)_2_S**. In contrast, after 48 h of proliferation, the ROS levels were significantly lower compared to the control and similar to those detected after 24 and 72 h of proliferation when cells were treated with 25 μN **(δ-Toc)_2_S_2_** and 50, 100, and 250 μN **δ-Toc** and its sulfur derivatives (Figure 8c).

### 2.7. Docking Calculations

In Table 1, we report the binding affinities (in kcal/mol) computed as described in the Section 4. Experimental binding affinities of 17β-E2 for ERα ds and ERβ were taken from Refs. [54] and [55], respectively. The experimental binding affinity of DPN vs. ERβ is taken from Ref. Handa 2022 [56]. The agreement between experimental and computed binding free energies for the estrogen receptor ligands with known affinities is satisfactory. In all cases, the best docking pose is remarkably close (less than 2 Angs. in terms of RMSD) to the corresponding crystallographic configuration, hence lending support to the Autodock-Vina calculations of the activity and binding structures of the tocopherol derivatives. The predicted binding free energy of **δ-Toc** for ERβ is −10.9 kcal/mol, a value that is comparable to that of the 17β-E2 substrate.

### 2.8. Binding Strength

For **(δ-Toc)_2_S** and **(δ-Toc)_2_S_2_**, we have used the 1HJ1 PDB structure for docking calculations. In the 1HJ1 co-crystal structure, the protein is missing the terminal H12 helix (partly sealing the LBD binding site in the apo form) due to the steric displacement of the anti-estrogen ICI-164,384, a synthetic derivative of estradiol bearing a long aliphatic chain [57,58]. The Vina-predicted dissociation constants of the **(δ-Toc)_2_S** and **(δ-Toc)_2_S_2_** bound to the H12-lacking ERβ 21 receptor are found in the sub-micromolar range as shown in Table 1, in agreement with their observed antiproliferation activity in HCT8-β8 cells (see Figure 3).

**Figure 9:**
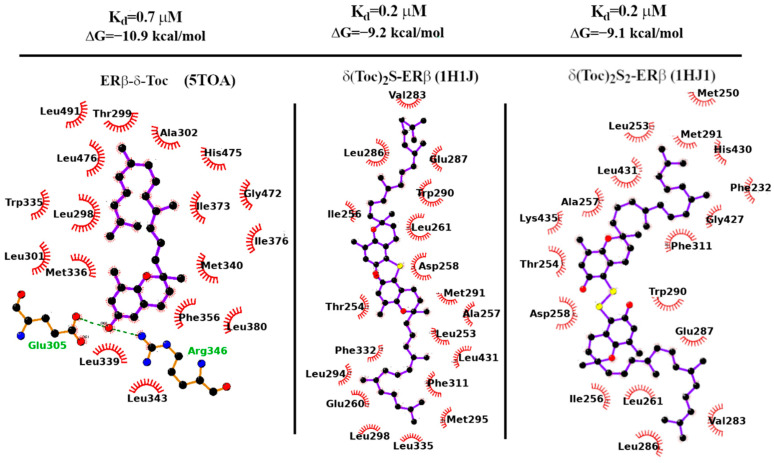
Binding affinity and dissociation constant of **δ-Toc**, **(δ-Toc)_2_S,** and **(δ-Toc)_2_S_2_** computed with Autodock-Vina 1.1.2 on the 5TOA and 1HJ1 X-ray structures of ERβ.

In Figure 9, we finally report the LigPlot-substituted tocopherol derivatives with ERβ [59]. In all cases’ diagrams for the mono and bi, the binding is overwhelmingly governed by non-polar contacts: out of the 19 contacts for **(δ-Toc)_2_S**, 15 are of the hydrophobic type. The same holds true for the **(δ-Toc)_2_S_2_** ligand where 14 out of 18 contacts involve hydrophobic side chains.

## 3. Discussion

The present study demonstrates, for the first time, the antiproliferative role of natural **δ-Toc**, and semi-synthetic **(δ-Toc)_2_S** and **(δ-Toc)_2_S_2_** in HCT8-β8 colorectal cancer cells overexpressing ERβ. The structural and functional analogy with 17β-E2, the lack of the antiproliferative effect in HCT8-β8 pretreated with ICI 182,780 and HCT8-pSV2neo cells, and the silico approach suggest that tocopherol-containing compounds may exert their antitumor function by binding to ERβ. Furthermore, similar to 17β-E2, the natural **δ-Toc** and semi-synthetic **(δ-Toc)_2_S** and **(δ-Toc)_2_S_2_** have been shown to upregulate the ERβ gene expression in HCT8-β8 only. In our experiments, the colon adenocarcinoma cell line, HCT8-β8, transfected to overexpress ERβ was used because the ERβ-mediated role of phytoestrogens or phenolic compounds in colon cancer has previously been investigated in this cell line [23,24], and colon cancer lines overexpressing ERβ have been used to study the anticancer role of this estrogen receptor [21].

The data obtained with **δ-Toc** and its sulfur derivatives were compared with the effect of 17β-E2, which has previously been shown to reduce proliferation, upregulate ERβ expression in HCT8-β8, and exert a protective role on colon mucosa to prevent the CRC [23,24,60]. Indeed, estrogens are able to regulate reproduction, signaling pathways and gene transcription in target tissues by binding to their nuclear receptor [1]. ERβ is the estrogen receptor that is predominantly expressed in the intestine, where it normally regulates the role of the epithelial barrier and the immune response. One of the roles of ERβ is to protect the colon from developing cancer, as a reduced expression of this receptor correlates with the risk and progression of CRC [13]. ERβ has been suggested to repress cancer-associated genes involved in cell proliferation and activate the expression of genes controlling cell growth inhibition, apoptosis, and differentiation [61]. Literature data suggest that various natural and synthetic compounds, such as phytoestrogens and xenoestrogens structurally related to estrogen, may play a protective role against CRC by binding to ERβ and enhancing its anticancer function [10]. In particular, isoflavones such as genistein and calycosin, and the selective agonist of ERβ, diarylpropionitrile, exert their anticancer properties by inhibiting proliferation and increasing apoptosis through ERβ in colon cancer cells [23,62,63].

**δ-Toc** and its sulfur derivatives, in addition to being structurally similar to 17β-E2, also exhibit an antiproliferative estrogen-like effect in HCT8-β8, but not in the presence of the ER antagonist ICI 182,780 nor in HCT8-pSV2neo, demonstrating that their effect on cell proliferation is mediated by the binding to ERβ. It is noteworthy that both **δ-Toc** and its sulfur derivatives achieve approximately similar maximal antiproliferative effects, but at different concentrations. In fact, this occurs with 250 μN **δ-Toc**, 50 μN **(δ-Toc)_2_S_2_**, and 12.5 μN **(δ-Toc)_2_S**, suggesting that sulfide/disulfide are able to activate the receptor at a lower concentration as compared to **δ-Toc**. All tocopherol-containing compounds bind to ERβ, as demonstrated by in silico simulations, but our results suggest that, because of their different chemical structure, they may have a different intrinsic activity and/or ability to induce the translocation of the receptor–ligand complex into the nucleus. In fact, in silico tests show that the affinity of **δ-Toc** for ERβ is greater than that of **(δ-Toc)_2_S** and **(δ-Toc)_2_S_2_**, but the antiproliferative effect of **δ-Toc** is much lower than that of its sulfur derivatives. This could be explained by the fact that the two sulfur compounds, which have two hydrophobic tails, give the receptor–ligand complex greater lipophilicity and facilitate its transfer into the nucleus. Moreover, although the sulfur derivatives of **δ-Toc** bind to the receptor with the same affinity, (**δ-Toc)_2_S_2_** has a lower ability to induce the receptor-mediated cellular response than **(δ-Toc)_2_S_,_** probably because the presence of the disulfide bond could alter the intrinsic ability of the derivative to activate the receptor and the nuclear translocation of the complex with ERβ**.**

Literature data show a direct interaction of tocopherols with ERα and ERβ, but **δ-Toc** is the component with the highest affinity able to increase the growth of the hormone-sensitive breast cancer cell MCF7, which expresses both ERα and ERβ [36]. **α-Toc** does not affect MCF7 cell proliferation, which is instead reduced by synthetic vitamin E derivatives [39], and it is not involved in ERβ nuclear translocation and the expression of ER-dependent genes in these cells [38]. Thus, the effect of vitamin E on cell proliferation depends on its different isoforms and ERα/ERβ ratio, which is high in breast cancer cells and low in colon cancer cells [64,65].

The downregulated expression of the *CCDN1* and *PLK 1* genes, resulting in a slowing of the cell cycle, is consistent with the antiproliferative properties of all compounds tested. A reduced expression of these genes was also observed in the antiproliferative role of the bioflavonoid luteolin in MCF7 cells [66].

**δ-Toc** and its sulfur derivatives at the concentrations tested have no effect on the cell viability of HCT8-β8 and HCT8-pSV2neo cells, and do not induce apoptosis in HCT8-β8 cells. On the contrary, 17β-E2 exerts a pro-apoptotic function in HCT8-β8 and reduces the viability in these cells but not in HCT8-pSV2neo cells. The effect of 17β-E2 on cell death is probably due to the ability of this hormone to influence biological mechanisms involved in cell death under certain conditions [67,68], and our results suggest that it is mediated through ERβ**.** Indeed, it has been shown that 17β-E2 is only able to induce apoptosis in Rat1 cells transfected to overexpress the ER receptor and not in parental Rat1 cells [69].

**δ-Toc, (δ-Toc)_2_S,** and **(δ-Toc)_2_S_2_** significantly increase the transcription of ERβ but not that of ERα in HCT8-β8. This increase in ERβ expression is consistent with what has been observed in the presence of 17β-E2 and phytoestrogens, such as genistein and quercetin, which not only reduce the proliferation but also upregulate the ERβ expression in colon cancer cells [23,70]. Moreover, the increase in ERβ expression is associated with an increase in ERβ protein levels, although a close relationship between ERβ and its mRNA levels in the presence of 17β-E2 and tocopherol-containing compounds was not found. This may be due to the fact that HCT8-β8 cells already overexpress the receptor and, therefore, not all of the mRNA is translated. In HCT8-pSV2neo, the treatment with 17β-E2, **δ-Toc,** and semi-synthetic sulfide and disulfide derivatives does not affect the expression of ERβ and ERα, suggesting that the increased transcription of ERβ is also mediated by the binding of compounds to ERβ.

We hypothesize that **δ-Toc** and the semi-synthetic sulfide and disulfide derivatives, by binding to ERβ and increasing its expression, modulate signaling pathways involved in the inhibition of cell proliferation. In fact, in breast cancer cells, calycosin inhibits cell proliferation by increasing ERβ expression, which induces changes in downstream signaling pathways, including the activation of p38 mitogen-activated protein kinase (MAPK) [71]. Bisphenol AF, an environmental contaminant, induces the apoptosis of human granulosa cells, KGN, through both ERβ and the ROS-dependent activation of MAPK pathways [72].

Indeed, ROS levels influence cell proliferation [73,74]. However, in our experimental conditions, starvation-induced oxidative stress is not related to the antiproliferative role of tocopherol-containing compounds considering that ROS levels are similar in both untreated and treated cells. **δ-Toc** and its sulfur derivatives have a low antioxidant capacity which is evident in HCT8-β8 cells only when a threshold is exceeded. In fact, after 48 h, δ**-Toc, (δ-Toc)_2_S,** and **(δ-Toc)_2_S_2_** are able to restore the levels of ROS to those found after 24 or 72 h of proliferation, but not to those of cells cultured in a complete medium (5% FBS). This is in contrast to what is observed in human colon adenocarcinoma epithelial cells, HT29, stimulated with TNFα. In this condition **δ-Toc, (δ-Toc)_2_S,** and **(δ-Toc)_2_S_2_** prevent TNFα-induced oxidative stress, and only δ-Toc exhibits pro-oxidant activity at high concentrations [75]. Moreover, 17β-E2, which attenuates oxidative stress in the starved osteocyte-like cell line MLO-Y4 and induces ROS production through ERβ activation in TCam-2 seminoma cells [76,77,78], does not affect starvation-induced ROS production in HCT8-β8 cells. These data demonstrate that the role of vitamin E, its sulfur derivative, and 17β-E2 on the intracellular redox state depends on the cell type and experimental conditions.

## 4. Materials and Methods

### 4.1. Cell Culture and Treatments

**Figure 10:**
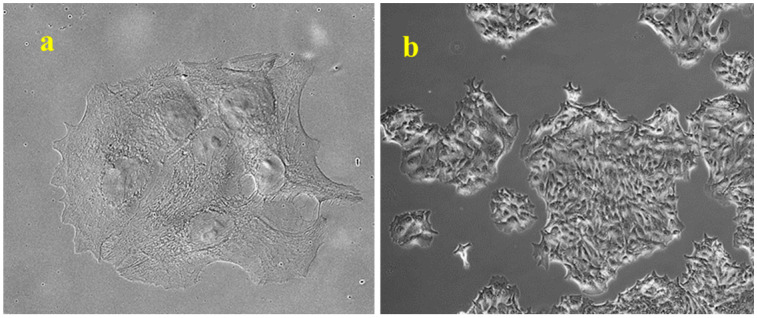
Representative observation of microscopic morphology of HCT8-β8 cells grown in RPMI 1640 with 10% FBS. (**a**) Cells observed in phase contrast, original magnification: 10×. (**b**) Cells observed in Nomarski differential interference contrast (DIC), original magnification: 63×.

Human colon adenocarcinoma HCT8 cells, obtained from the American Type Culture Collection (Rockville, MD, USA), were stably transfected with the mammalian expression vector pCXN2-hERβ (HCT8-β8) or a control pSV2neo vector (HCT8-pSV2neo) [22]. Similar to HCT8-pSV2neo, HCT8-β8 cells exhibited a polygonal shape with few extensions and a well-defined cytoskeleton when cultured in RPMI 1640 medium (Lonza Group, Basel, Switzerland) with 10% FBS; they grow in islets before reaching a packed confluence of approximately 3 × 10^5^ cells/cm^2^ (Figure 10).

Cells were cultured at 37 °C in a humidified atmosphere of 5% CO_2_ in RPMI-1640 medium supplemented with 1 mmol/L sodium pyruvate, 2 mmol/L L-glutamine, 100 μg/mL penicillin, 100 μg/mL streptomycin, 800 μg/mL di geneticin (G418; Invitrogen from Thermo Fisher Scientific Inc., Waltham, MA USA) (growth medium), and 10% FBS. The cells were then seeded at a density of 0.35 × 10^3^ cells/cm^2^ in dishes or multiwell plates, as appropriate for the experiments, and cultured in growth medium supplemented with 5% FBS (complete medium). After 72 h, the complete medium was replaced with growth medium with 0.5% charcoal-stripped FBS (Biological Industries, Kibbutz Beit Haemek, Israel) (starved medium) for 24 h (0 h). Subsequently, cells were cultured in starved medium and treated with 10 nM 17β-E2, used as a control for antiproliferative effect, or different concentrations (12.5–250 μN) of **δ-Toc** and the semisynthetic, **(δ-Toc)_2_S** and **(δ-Toc)_2_S_2_**, for further appropriate time periods (h).

Solutions of **δ-Toc**, **(δ-Toc)_2_S**, **(δ-Toc)_2_S_2_**, and 17-β-E2 in ethanol were diluted in starved medium in order to achieve the required concentration. Then, 1‰ ethanol was added to the respective untreated cells. All reagents used in our experiments were purchased from Sigma-Aldrich (St. Louis, MO, USA), unless otherwise noted.

### 4.2. Bis-δ-Tocopheryl Sulfide (δ-Toc)_2_S and bis-δ-Tocopheryl Disulfide (δ-Toc)_2_S_2_ Synthesis

Bis-δ-tocopheryl disulfide **(δ-Toc)_2_S** and bis-δ-tocopheryl disulfide **(δ-Toc)_2_S_2_** were synthesized as previously described [44,79].

### 4.3. Cell Proliferation Analysis

Proliferation experiments were performed in cells seeded in a 60 mm tissue culture dishes as described above. HCT8-β8 cells were grown in starved medium for 24 h (0 h) and then treated or not with the 17β-E2 or tocopherol-containing compounds for a further 24, 48, and 72 h (proliferation times). In other experiments, test compounds were added to HCT8-β8 pretreated for 1 h with 10 μM ICI 182,780 (fulvestrant) (Merck KGaA, Darmstadt, Germany). Cells were detached with a trypsin/EDTA solution and counted using a Bürker hemocytometer (Labor Optik, Lancing, UK) at the indicated times during the logarithmic phase of cell growth to avoid the contact inhibition phenomenon. Cell counts were plotted as linear regressions with log_10_ of cell number on the *y*-axis and time on the *x*-axis, and results were expressed numerically as population-doubling time in h as mean ± SE.

### 4.4. Colony Formation Assay

Cells cultured for 72 h in complete medium were detached and seeded in 6-well plates at a density of 25, 50, 100, and 200 cells/well. After 12 h, the time required for cells to attach to the wells, complete medium was replaced with starved medium and 10 nM 17β-E2, 250 μN **δ-Toc**, 12.5 μN **(δ-Toc)_2_S,** and 50 μN **(δ-Toc)_2_S_2_** were added for 10 days to HCT8-β8 pretreated or not with 10 μM ICI 182,780 or to HCT8-pSV2neo. The colonies formed were fixed with 4% paraformaldehyde for 10 min, washed three times with H_2_O, and stained with 1% toluidine blue for 5 min. The colonies were then washed three times with H_2_O and counted with a stereomicroscope WILD M3 (WILD Heerbrugg AG, Heerbrugg, Switzerland) at 6.4× magnification. Only colonies with more than 50 cells were included in the analysis. Images were acquired using LSM-900 confocal microscope (Carl Zeiss, Ober-kochen, Germany) in brightfield with 2.5 magnification at a density of 200 seeded cells per well by panning, and each image is derived from the sum of a composition of an average of 64 individual images.

### 4.5. Cell Viability Analysis

Cell viability was determined in HCT8-β8 and HCT8-pSV2neo seeded in 24-well plates and treated as described above. Lysosome formation was detected by AO staining in cells cultured in complete medium, to verify the absence of lysosomal activity, or in starved medium and untreated (control) or treated with 10 nM 17β-E2, 250 μN **δ-Toc**, 12.5 μN **(δ-Toc)_2_S,** and 50 μN **(δ-Toc)_2_S_2_** for 24, 48, and 72 h. The cells were washed three times with phosphate-buffered saline (PBS) and incubated in 1.5 × 10^−5^ Mol/L AO solution in PBS for 5 min in the dark at room temperature. Then, after three washes with PBS, starved medium was added to the cells to prevent cell damage during microscopic observation.

Cells were observed with an LSM-900 confocal microscope (Carl Zeiss, Oberkochen, Germany) using laser excitation at 488 nm (λem 495–560 nm) to stain cytoplasm and nucleus with orthochromatic green fluorescence and laser excitation at 405 nm (λem 575–700 nm) to stain lysosomes with metachromatic red fluorescence. Images were acquired using ZEN 3.1 software (Zeiss). Lysosomal activity was quantified by the red/green fluorescence ratio for each treatment and data were expressed as percentage of the control.

For the trypan blue exclusion test, cells were detached and a small volume of cell suspension was mixed with an equal volume of trypan blue dye. Dead (stained) and live (unstained) cells were counted in a Burker hemocytometer. The ratio of the number of unstained cells to the total number of cells × 100 was used to express the percentage of viable cells.

### 4.6. Cell Apoptosis Assay

HCT8-β8 were seeded in 24-well plates and treated or not with 10 nM 17β-E2, 250 μN **δ-Toc**, 12.5 μN **(δ-Toc)_2_S**, 50 μN **(δ-Toc)_2_S_2_**, and 1 μM ionomycin for 72 h as described above. Apoptosis was detected by using Cell Death detection ELISAplus kit (Sigma-Aldrich, St. Louis, MO, USA) according to the manufacturer’s instruction. Mono- and oligonucleosomes released from 10^4^ cells were detected using two monoclonal antibodies directed against DNA and histones and data are expressed as fold increase over controls using the following ratio: absorbance of sample/absorbance of control.

### 4.7. RNA Isolation and Real-Time qPCR

Total RNA was isolated from cells seeded in 6-well plates and treated or not with 10 nM 17β-E2, 250 μN **δ-Toc**, 50 μN **(δ-Toc)_2_S,** and 50 μN **(δ-Toc)_2_S_2_** for 24 h of proliferation as described above. RNA was isolated using Qiazol Lysis Reagent (Qiagen, Hilden, Germany) according to the manufacturer’s instructions and quantified by UV absorbance. Reverse transcription was performed using the Quantitect Reverse-Transcription Kit followed by treatment with ribonuclease-free deoxyribonuclease I (Qiagen). Quantitative polymerase chain reaction (qPCR) was performed using the Kapa Probe Fast qPCR kit (Kapa Biosystems Inc., Wilmington, MA, USA) according to the manufacturer’s instructions. Briefly, reactions consisting of 1 μL cDNA, 10 μL KAPA PROBE FAST qPCR Master Mix, 2 μL gene specific primers (10 μmol/L), 1 μL TaqMan Probe (5 μmol/L), and 6 μL RNase-free H_2_O were heated at 95 °C for 5 min and amplified by 35 cycles of 95 °C for 10 s, and 60 °C for 30 s using a Rotor-Gene Q (Qiagen). The results obtained were normalized to the ribosomal protein S1 (RPS18) housekeeping gene (RPS18). The following primers and Taq-Man probes were used as listed in Table 2.

### 4.8. Immunofluorescence Staining of ERβ

HCT8-β8 were seeded in 24-well plates and treated or not with 10 nM 17β-E2, 250 µN **δ-Toc**, 12.5 µN **(δ-Toc)_2_S,** or 250 µN **(δ-Toc)_2_S_2_** as described above. After 48 and 72 h of treatment, cells were fixed for 10 min with 4% paraformaldehyde and permeabilized with 0.2% Triton 100× at 37 °C for 30 min in humidified air with 5% CO_2_. After three washes with PBS, the cells were treated with RNase diluted 1/1000 with 2% bovine serum albumin (BSA)/PBS at 37 °C in humidified air with 5% CO_2_ to block non-specific sites and degrade RNA. Cells were washed three times with PBS and were incubated overnight at 4 °C with the primary antibody for ERβ (Cell Signaling, Danvers, MA, USA). After removal of the primary antibody with three washes with PBS, the secondary antibody (Goat anti-Rabbit IgG (H + L) superclonal secondary antibody, Alexa Fluor 488, Invitrogen (Thermo Fisher Scientific, Waltham, MA, USA) was added in the dark for 45 min at room temperature. The cells were washed with PBS and the nuclei were counterstained with propidium iodide for 5 min at room temperature. After washing with H_2_O, the cells were observed by laser scanning confocal microscopy (LSM 900, ZEISS) at 63×.

### 4.9. ERβ Protein Assay

ERβ protein level expression was detected in cell lysates from HCT8-β8 seeded in 12-well plates and treated with 10 nM 17β-E2, 250 μN **δ-Toc**, 50 μN **(δ-Toc)_2_S,** and 50 μN **(δ-Toc)_2_S_2_** for 48 and 72 h as described above, using a Human ERβ ELISA kit (Elabscience, Houston, TX, USA), according to the manufacturer’s instructions. Briefly, cells were detached with trypsin and centrifuged for 5 min at 1000× *g*. The pellet containing the cells was washed three times, resuspended in cold PBS, and sonicated until the cells were completely lysed. The cell lysates were then centrifuged at 1500× *g* for 10 min at 4 °C to remove cell fragments and the supernatants were used for the assay. Data, normalized on total protein content, were expressed as a percentage of the ERβ level detected in the control.

### 4.10. Intracellular ROS Assay

Intracellular ROS production was measured, as previously described [80], in HCT8-β8 cells seeded in 12-well plates and treated as described above. Specifically, the ROS assay was performed in HCT8-β8 cells cultured in complete medium, and in starved medium for 24 h (0 h). Additionally, ROS were detected at proliferation times in cells cultured in starved medium and treated or not with 10 nM 17β-E2 or tocopherol-containing compounds at all concentrations used. Cell permeant 2′-7′-dichlorodihydrofluorescein diacetate (H2DCF-DA, Invitrogen, Carlsbad, CA, USA) is a fluorogenic compound that is deacetylated inside cells to a non-fluorescent compound. Upon oxidation by ROS, this compound is converted to the fluorescent compound 2′-7′-dichlorodihydrofluorescein. Then, 5 mg/L H2DCF-DA was added to the cells 30 min before the end of each treatment. HCT8-β8 cells were lysed in a buffer containing 50 mM Tris/HCl pH 7.5, 1% Triton X-100, 150 mM NaCl, 100 mM NaF, and 2 mM EGTA, after washing with PBS. Lysed cells were centrifuged at 10,000× *g* for 10 min and fluorescence was detected in a Fluoroskan AscentFL microplate reader (Thermo Fisher Scientific, Waltham, MA, USA) at an excitation wavelength of 485 nm and an emission wavelength of 518 nm. Data were normalized to total protein content and expressed as percent ROS measured in complete medium or ROS at 0 h.

### 4.11. Protein Assay

Bicinchoninic acid solution (BCA) protein reagent assay (Thermo Fisher Scientific, Waltham, MA, USA) and BSA as standard were used to determine protein concentration as previously described [81].

### 4.12. In Silico Approach for the Prediction of the Binding of Tocopherols to ERβ

The 3D structure of the Ligand Binding domain (LBD) of human ERβ in complex with diarylpropionitrile (DPN) was obtained from the PDB files 7XVY [48], 1HJ1 [82], and 5TOA in complex with estradiol [83]. The 3D structure of the Ligand Binding domain (LBD) of human ERα in complex with diethylstilbestrol (DSB) was obtained from the PDB file 3ERD [84], and PDB file 1A52 in complex with estradiol [85]. The 3D structure of the compounds (**δ-Toc)_2_S, (δ-Toc)_2_S_2_**, **α-Toc**, **and δ-Toc** were generated using the OpenBabel suite from the corresponding isomeric SMILES codes [86]. The docking calculations were performed using the Autodock-Vina code assuming a rigid receptor for the substrate 17β-E2 and for the ligands (R)DPN and DSB [87]. For the bulky tocopherol derivatives, the residues GLU353, LEU384, LEU391, ARG394, LEU424, and LEU525 of ERα, and the residues GLU305, MET336, LEU339, PHE356, ILE376, HIS475, and LEU476 of ERβ were assumed to be flexible. Flexible residues have been selected by analyzing the best docked pose of the substrate 17β-E2 with ERα/β, as those amino acids having at least one atom of the side chain within 3.8 Å of any atom of 17β-E2. When using a rigid receptor, for the docking calculation, we retained the default Autodock setting (40 × 40 × 40 points with a grid spacing of 0.375 Å, yielding a cubic box of 15 Å, and 50 minimization rounds). When dealing with a flexible receptor, as for the case of the tocopherol derivatives, we used 60 × 60 × 60 points with a grid spacing of 0.375 Å, with a cubic box of 22.5 Å, and 70 minimization rounds. The cubic box was centered in all cases at the center of mass of 17β-E2 in the best docking pose.

### 4.13. Statistical Analysis

As a first step, the normality of the replicates and the homoscedasticity of the groups were verified using the Lilliefors and Levene tests, respectively.

The statistical significance of the differences between the media in cell viability, cell apoptosis, RT-qPCR, ERβ and ERα proteins, and intracellular RO assays was determined by one-way ANOVA analysis and by post hoc Bonferroni’s multiple comparison test with a predefined experimental probability α_T_ = 0.05 using GraphPad Prism10 software (GraphPad Software, San Diego, CA, USA).

Statistical analysis of cell proliferation and colony formation was performed as follows: (a) goodness-of-fit of each linear regression plot was verified by linearity test carried out using one way ANOVA, and (b) statistical differences between treated groups and control were analyzed by parallelism test performed in Excel by one-way ANOVA followed by post hoc Bonferroni’s multiple-comparison test with a predefined experimental probability α_T_ = 0.05.

## 5. Conclusions

This study demonstrates, for the first time, that natural **δ-Toc** and semisynthetic δ-tocopheryl sulfide **(δ-Toc)_2_S** and disulfide **(δ-Toc)_2_S_2_**, similar to 17β-E2, are able to reduce proliferation and increase the ERβ expression in human colon adenocarcinoma HCT8 cells engineered to overexpress ERβ but not in HCT8-pSV2neo. In addition, the lack of the antiproliferative effect of the compounds tested in HCT8-β8 treated with ICI 182,780 and the in silico assay support the hypothesis that **δ-Toc, (δ-Toc)_2_S,** and **(δ-Toc)_2_S_2_** act through their binding to ERβ. No involvement of the intracellular redox state in the antiproliferative role of the compounds was demonstrated. All tocopherol-containing compounds at the concentrations tested are not pro-apoptotic and do not affect cell viability but downregulate the expression of genes involved in cell cycle regulation. The results obtained suggest that both **δ-Toc** and its sulfur derivatives may play a role preventive in colorectal cancer through ERβ. However, **(δ-Toc)_2_S** and **(δ-Toc)_2_S_2_** are able to exert their protective effect at lower concentrations. Taken together, these data may provide the basis for new anticancer therapeutic strategies. Further studies are needed to identify the signaling pathways involved in the ERβ-mediated antiproliferative effects of tocopherol-containing compounds.

## Figures and Tables

**Table 1:** Binding free energies (in Kcal/mol) for various ERα and ERβ ligands (see text) predicted using Autodock-Vina.

		ERα			ERβ	
**Ligand**	**PDB**	**ΔG_calc_**	**ΔG_exp_**	**PDB**	**ΔG_calc_**	**ΔG_exp_**
17β-E2	1A52	−10.2	−13.4	5TOA	−10.5	−13.2
DPN	−	−	−	7XVY	−8.7	−9.0
**δ-Toc**	3ERD	−8.4	−	7XVY	−10.9	−
**(δ-Toc)_2_S**	−	−	−	1HJ1	−9.2	−
**(δ-Toc)_2_S_2_**	−	−	−	1HJ1	−9.0	−

− no docking calculations or no experimental binding affinity are available.

**Table 2:** Primers and TaqMan used for quantitative real-time PCR (RT-qPCR).

Gene	Oligonucleotides	Sequence (5′ → 3′)	Amplicon Size (bp)	T_ann_ (°C)
ERβ	Forward primer Reverse Probe *	TCGCCAGTTATCACATCTGTATGCGG GTGTCTCTCTGTTTACAGGTAAGGTGTG **F**TCCCGGTG**Z**TGAAGCAAGATCGCTAGAA**Q**	95	65
ERα	Forward primer Reverse Probe *	GACTATGCTTCAGGCTACCATTA GGCTGGACACATATAGTCGTTAT **F**TCTCTTGAA**Z**GAAGGCCTTGCAGCC**Q**	120	60
CCND1	Forward primer Reverse Probe *	CTGTGCATCTACACCGACAA AGGTTCCACTTGAGCTTGTT **F**AGCTCCATT**Z**TGCAGCAGCTCCTC**Q**	86	55
PLK1	Forward primer Reverse Probe *	CACAGTGTCAATGCCTCCAA AGGCCGTACTTGTCCGAATA **F**ATCTTCTGG**Z**GTCAGCAAGTGGGTG**Q**	125	55
RPS18	Forward primer Reverse Probe *	AATCCGTTGACTCCGACCTTC ACAGTACAGCCGCATCTTC **F**CCACATCGC**Z**TCAGACACCATGGG**Q**	179	69

* TaqMan probes in which **F**, the reporter fluorochrome [6-carboxyfluorescein], **Z**, the internal ZEN quencher, and **Q**, the quencher fluorochrome (Iowa BlacK FQ), are present. bp, base pairs of amplicon size; T_ann_, annealing temperature.

## Data Availability

The datasets analyzed in the current study are not publicly accessible, but they are available from the corresponding author upon reasonable request.
